# Trust matters: The Addressing Vaccine Hesitancy in Europe Study

**DOI:** 10.1177/14034948231223791

**Published:** 2024-02-12

**Authors:** Pia Vuolanto, Ana Nunes Almeida, Alistair Anderson, Petra Auvinen, André Beja, Piet Bracke, Mario Cardano, Melissa Ceuterick, Tiago Correia, Elisabetta De Vito, Katrijn Delaruelle, Ana Delicado, Maurizio Esposito, Maria Ferrara, Luigi Gariglio, Cátia Guerreiro, Jaroslava Hasmanová Marhánková, Ana Patrícia Hilário, Pru Hobson-West, Juliana Iorio, Katri-Maria Järvinen, Annariina Koivu, Zuzana Kotherová, Aapo Kuusipalo, Esther Lermytte, Joana Mendonça, Rita Morais, Dino Numerato, Paulina Polak, Tadeusz Rudek, Sara Sbaragli, Alice Scavarda, Katielle Silva, Pedro Alcantâra da Silva, Jonas Sivelä, Eva Soares Moura, Maria Świątkiewicz-mośny, Giuseppe Tipaldo, Aleksandra Wagner

**Affiliations:** 1 Tampere University, Faculty of Social Sciences, Research Center for Knowledge, Science, Technology and Innovation Studies, Finland; 2 Universidade de Lisboa, Instituto de Ciências Sociais, Portugal; 3 University of Nottingham, School of Sociology and Social Policy, UK; 4 Global Health and Tropical Medicine, GHTM, Associate Laboratory in Translation and Innovation Towards Global Health, LA-REAL, Instituto de Higiene e Medicina Tropical, IHMT, Universidade Nova de Lisboa, UNL, Portugal; 5 Ghent University, Department of Sociology, Health and Demographic Research, Belgium; 6 University of Turin, Department of Cultures, Politics and Society, Italy; 7 University of Cassino and Southern Lazio, Department of Human, Social and Health Sciences, Italy; 8 Charles University, Faculty of Social Sciences, the Czech Republic; 9 Jagiellonian University, Institute of Sociology, Poland; 10 Finnish Institute for Health and Welfare, Cultural, Behavioural and Media Insights Center, Finland

**Keywords:** Vaccine hesitancy, health sociology, public health, cross-country study, study design

## Abstract

This article presents the design of a seven-country study focusing on childhood vaccines, Addressing Vaccine Hesitancy in Europe (VAX-TRUST), developed during the COVID-19 pandemic. The study consists of (a) situation analysis of vaccine hesitancy (examination of individual, socio-demographic and macro-level factors of vaccine hesitancy and analysis of media coverage on vaccines and vaccination and (b) participant observation and in-depth interviews of healthcare professionals and vaccine-hesitant parents. These analyses were used to design interventions aimed at increasing awareness on the complexity of vaccine hesitancy among healthcare professionals involved in discussing childhood vaccines with parents. We present the selection of countries and regions, the conceptual basis of the study, details of the data collection and the process of designing and evaluating the interventions, as well as the potential impact of the study. Laying out our research design serves as an example of how to translate complex public health issues into social scientific study and methods.

## Background

This paper presents the research protocol of the Addressing Vaccine Hesitancy in Europe (VAX-TRUST) study, running from March 2021 until February 2024 and funded by the European Union’s Horizon 2020 research and innovation programme. Carried out by sociologists and public health scholars, VAX-TRUST analyses vaccine hesitancy as a complex transnational, yet region- and context-specific phenomenon in today’s welfare societies, namely Finland, Belgium, Poland, Italy, Portugal, the Czech Republic and the UK. Our specific focus was on childhood vaccines. We concentrated on situations where healthcare professionals (HCPs) engage with parents and explored what happens during vaccination visits because this may impact vaccine hesitancy.

Vaccine hesitancy as a term captures a dynamic spectrum of engagements with vaccines, ranging from the complete refusal of all vaccines, the refusal of vaccines but hesitant about this decision, hesitating about some vaccines or only one of them, to hesitating but still taking vaccines [1,2]. Vaccine hesitancy was recognised as a global health threat by the World Health Organization (WHO) before the COVID-19 pandemic [3]. The emergence of the COVID-19 pandemic accentuated the issue of vaccine hesitancy as countries across the globe realised in a renewed way the extent to which individuals may be hesitant towards vaccines and discussions about low childhood vaccination rates became intertwined with discussions about COVID vaccine uptake [4]. However, vaccine hesitancy has been observed since the development of vaccines and appears especially with recently approved and childhood vaccines, but also with vaccines that have been in use for a longer period of time [5,6]. Low vaccine rates appear across the globe due to poor access to immunisation services, but vaccine hesitancy is especially an issue in parts of Europe, where vaccine rates continue to be lower than might be expected despite the availability of services [7].

This paper shows how to translate complex public health issues into social scientific research across different country and healthcare system contexts. The aim of VAX-TRUST was to (a) conduct social scientific and context-sensitive research on vaccine hesitancy in specific regions, (b) support HCPs in their engagements with vaccine hesitancy and (c) draw recommendations for addressing vaccine hesitancy on different policy levels. Social scientific knowledge has been considered important in understanding parents' reasons for vaccine hesitancy and how to respond to their concerns, as well as in gaining a better understanding of the position and attitudes of HCPs themselves when encountering vaccine-hesitant individuals [8,9]. With VAX-TRUST, we aimed to analyse the role of HCPs and to provide them with tailored, region-specific and evidence-based knowledge. VAX-TRUST may help HCPs to recognise societal and cultural aspects of vaccine hesitancy.

## Selection of countries and regions

Seven European countries were identified. These were selected as representing a diversity of healthcare system characteristics, vaccine policy and immunisation infrastructure, regulatory environments, epidemiological considerations, cultural, socio-demographic, and geographical diversity, and previous research and data availability in each country.

**Figure 1.**
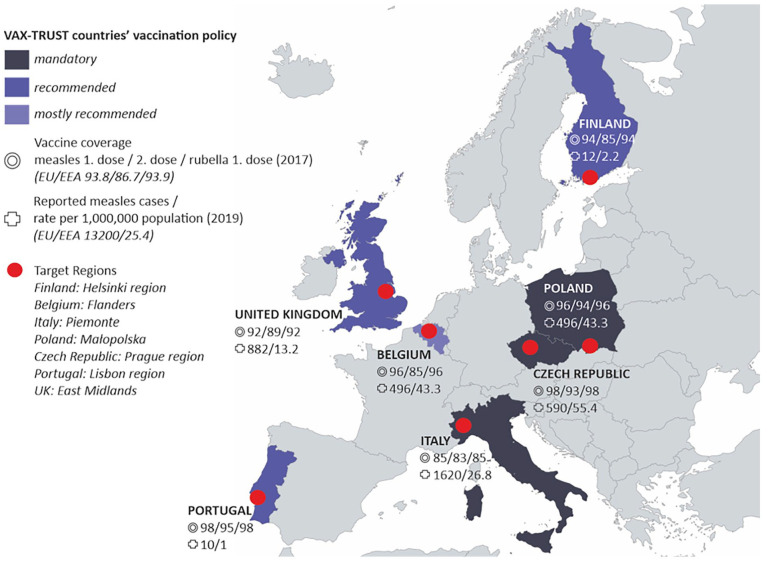
VAX-TRUST countries and target regions.

VAX-TRUST was designed to focus on a range of European countries that differ in size and include those with mandatory childhood vaccine policies and those where some or all childhood vaccines are voluntary. The childhood vaccine coverages are significantly lower in some countries than in others, as exemplified with measles and rubella immunisation coverage and measles incidence rates in Figure 1.

In brief, Finland is a small Nordic country where voluntary and free childhood vaccinations are available through child health clinics. The mid-size central European country of Belgium offers a perspective of a country where the vaccination programme falls under the jurisdiction of different communities and where only one of the childhood vaccinations (polio) is compulsory. Poland is a large central–eastern European country where healthcare is based on a system of mandatory insurance and where private, mainly out-of-pocket spending constitutes a major part of the healthcare system. Some vaccinations are mandatory in Poland, while others are recommended. The large southern European country of Italy makes an interesting case study because a mandatory vaccination policy for school admission was introduced in 2017. Portugal represents a mid-size southern European country with relatively high rates of vaccination and high vaccination confidence, where vaccination is universal, free and accessible to all population. The Czech Republic, a mid-size country in central Europe, offers a case where the healthcare system is based on compulsory statutory health insurance, immunisation of children is mandatory for most childhood vaccinations, and refusal can be fined and lead to exclusion from pre-school education. A western European country, the UK represents a large, nationalised healthcare system where vaccines are recommended and administered to the public via doctors’ surgeries. The UK has a rich history in relation to childhood vaccine debates, particularly around the MMR (measles, mumps and rubella) vaccine.

Within these countries, we have selected specific regions referred to as target regions (Figure 1). The selection of target regions was based on the fact that there had recently been outbreaks of vaccine-preventable diseases in the region, indicating that HCPs in these regions meet with vaccine-hesitant parents in their everyday encounters.

## Conceptual basis of VAX-TRUST

Vaccine hesitancy, as all complex societal phenomena, happens in certain places and situations and we designed VAX-TRUST to acknowledge and respect this socio-cultural complexity. Four specific assumptions have guided our research initiative.

- The importance of placing vaccine hesitancy within a social and cultural context. Much previous research has devoted attention to the individual-level determinants of vaccine hesitancy [10–12]. However, attitudes towards vaccines may additionally be shaped by the societal conditions and socio-cultural context where citizens are embedded: citizens with a specific socio-demographic profile in certain countries can have more positive attitudes towards vaccines than citizens with a similar profile from other countries. With this notion, we sought to highlight that health behaviour and health decision-making do not take place in a vacuum, also acknowledging the possible intervening role of factors such as institutional and societal trust, general degree of corruption, unemployment rates or a broader role of healthcare systbibrems. Acknowledging these allows for the development of public health interventions that are not only scientifically sound, but also culturally sensitive, and ultimately, more effective in their goals.
- The importance of public debates in the mass and social media. Social and mass media form an increasingly leading source of health-related information, not only for the general public, but also for HCPs [13]. While it is not the case that media discourses are the only factor, all actors are ex-posed to various vaccine discourses in the mass and social media or online public sphere more broadly [14,15], HCPs therefore need to be cognisant of the various vaccine discourses that surround their patients and themselves. This allows them to frame messages in ways that consider the prevailing narratives, empathically interact with vaccine-hesitant individuals as well as better un-derstand their own possible hesitancy.
- The importance of seeing vaccine hesitancy as a relational phenomenon. Previous research has primarily focused either on the individuals’ reasons not to vaccinate or on HCPs’ attitudes towards vaccinations. In other words, the focus has often been on the characteristics of each stakeholder. By contrast, we highlight vaccine hesitancy as a relational phenomenon [8] and thus emphasise the relationships between the main actors in childhood immunisation activities: HCPs, parents and children. Normally, vaccines are given in a situation where these worlds meet. All worlds bring to the vaccine encounter, among other issues, their values, lifestyles and experiences. Focusing on the encounter between vaccinating HCPs and parents with children to be vaccinated, VAX-TRUST highlights the central role of trust in the interaction [8,16]. HCPs thus need to ensure sensitivity to the lifeworld of parents and children, but also be supported to reflect on their own values and experiences of vaccination, recognising the fact that HCPs may be vaccine-hesitant themselves. This assumption emphasises that even though levels of vaccine hesitancy are not only dependent on the encounters between HCPs, parents and children, but shaped by the socio-cultural factors and societal debates as well, the role of HCPs is fundamental in building or sustaining trust towards expertise, the healthcare system and evidence-based recommendations.
- The significance of fostering dialogue and constructive engagement in the situations where vaccination is being discussed or administered. Previous attempts to address vaccine hesitancy have been either on focusing on parents or on improving HCPs’ confidence and communication skills, or they have been targeted at the community level [17]. VAX-TRUST attempts to further a two-way dialogical process in immunisation and to consider the different perceptions about vaccination of these actors. We are focused on understanding the good reasons [18] of the parties in the debate: (a) listening carefully to the vaccine concerns and sceptical voices of vaccine-hesitant parents [9] and to avoid blaming hesitant parents for their ‘ignorance’, failure to understand science or for being against science [8]; and (b) avoiding blaming HCPs for doing something wrong or oversimplifying the issue as poor communication [19]. Indeed, through its multidisciplinary, inclusive study design capturing a broad range of experiences from both parties, and through the provision of training which aims to support respectful conversations with hesitant parents, VAX-TRUST aims to build bridges between HCPs and parents.

**Figure 2.**
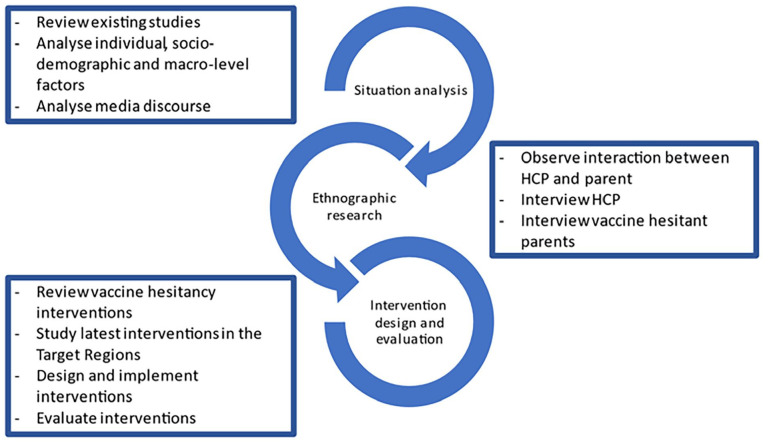
The stages of VAX-TRUST.

In addition to these four assumptions, we considered the WHO guidelines for tailoring immunisation programmes, which suggest that to understand the phenomenon of low vaccine uptake fully, and to design sustainable solutions to address it, requires careful situation analysis, in-depth research in the context, and thorough intervention design and implementation [20]. Reflecting these steps, VAX-TRUST focuses on (a) producing an overview of existing evidence in the form of situational analysis of vaccine hesitancy in Europe (VAX-TRUST situation analysis), (b) conducting ethnographic research for novel insights into vaccine encounters (VAX-TRUST ethnographic research) and (c) designing and implementing an evidence-based intervention (VAX-TRUST intervention design and evaluation) (Figure 2). These phases form the basis of evidence-based VAX-TRUST recommendations to the European, national and local public health authorities. In addition to these research components, the project includes components focusing on ethics, management and dissemination of project results.

## VAX-TRUST situation analysis

VAX-TRUST research for situation analysis aims at increasing knowledge about vaccine hesitancy in specific regions through review of existing studies regarding vaccine hesitancy, analysis of macro-level factors impacting vaccine hesitancy, analysis of individual and socio-demographic factors of vaccine hesitancy and resistance, and analysis of media coverage on vaccinations. The situation analysis includes the following components and respective data sources: (a) examination of individual, socio-demographic and macro-level factors of vaccine hesitancy (literature review and survey data) and (b) analysis of media coverage (major news portals and websites of societal groups and organisations focusing on the negative effects of vaccination).

Within the situation analysis, the first part utilised pre-existing quantitative survey data (Eurobarometer 91.2) combined with information retrieved from several public datasets [21–23]. These data were chosen because they included diverse questions on vaccine attitudes and enabled examination of the relationship between attitudes and macro-level factors (see Table I). The second part of the situation analysis gathered and quantitatively and qualitatively analysed media data (Table I; see Polak et al. [24] for further details). Major news portals were chosen to find similarities and differences in the mainstream vaccination discourses within the seven countries. Websites of societal groups and organisations dealing with negative effects of vaccination were used to compare with discourses that counter or question mainstream discourses.

**Table I.** Research objectives, data sources and methods for VAX-TRUST situation analysis.

Research objectives	Data	Methods	Detailed methods
Review existing evidence on vaccine hesitancy from seven European countries to summarise existing information and identify gaps in knowledge on vaccine hesitancy in VAX-TRUST countries and target regions	Academic and policy literature	Content analysis of literature	Review of national reports, recommendations and assessments, strategies and action plans for immunisation, and academic publications on vaccine hesitancy
Analyse individual, socio-demographic, and macro-level factors of vaccine hesitancy prior to the COVID-19 pandemic	Eurobarometer 91.2 data (27,524 individuals, 28 countries) and other data sources for the macro-level indicators	Survey questions included: ‘It is important for everybody to have routine vaccinations’; ‘Vaccines are only important for children’; ‘Not getting vaccinated can lead to serious health issues’; ‘Vaccines are important to protect not only yourself but also others’; ‘Vaccination of other people is important to protect those that cannot be vaccinated’; and ‘Do you think that vaccines can be effective in preventing infectious diseases?’	Examining the relationship between people’s attitudes toward vaccination and macro-level factors. a. Vaccination programmes (i.e. the organisation of vaccination services, the provision of vaccination services and the financing of vaccination services) b. Vaccination coverage rates (up to date from national registries) c. Past disease exposure (data from the *Surveillance Atlas of Infectious Diseases*) d. Broader healthcare system characteristics (e.g. density of healthcare providers) e. General societal characteristics (e.g. the level of corruption, trust in healthcare, trust in science) Focus of analysis: impact of socio-demographic factors (e.g. gender, age, educational status, occupational status, marital status and political orientation) on vaccine hesitancy according to the macro-level factors. Analysis methods: univariate, bivariate and multivariate analyses combined with information retrieved from several public datasets; composite indicators (ANOVA and χ^2^ tests), multilevel regression modelling [25]
Conduct analysis of media coverage to understand societal discussion on vaccines and vaccination before and during the COVID-19 pandemic	Articles in mainstream news portals (websites of national broadcasting companies, major newspapers and nationwide tabloids); total in the seven VAX-TRUST countries *n*=47,845	Analysis in a two-step procedure: (a) quantitative (text mining); and (b) qualitative discourse analysis of media discourse, with the use of a common codebook	Analysis of: a. The main discursive threads in the media discussions, their public visibility understood as a media exposition and their development in time, with particular focus on the periods of outbreaks, vaccine uptake decreases or COVID-19 pandemic b. The visible and invisible actors of the societal discussions as well as the part played by HCPs and healthcare authorities in the discussions c. The types of reasoning and argumentation constructed via media discourse and their mutual interplay
	One to three websites of societal groups and organisations dealing with negative effects of vaccination per country	Qualitative discourse analysis	Mapping the discussions in the hesitancy arenas that counter or question the necessity, safety or reasonableness of vaccination

## VAX-TRUST ethnographic research

VAX-TRUST conducted research on the interactions between HCPs and parents (in-depth interviews and observation data). The objective was to conduct qualitative research on these interactions to gain novel insights into vaccine encounters. We aimed at understanding the effects of the interaction between parents and HCPs on parental attitudes towards vaccination and the ways HCPs encounter vaccine hesitancy in their everyday contexts of practice. We chose a qualitative approach as it fits best with our conceptual commitments to understanding the worlds of both parents and professionals. Many previous studies on vaccine hesitancy have focused either on parents [26,27] or HCPs [28–32] and thus the observation of their interaction in a clinical setting represents a novel methodological approach. This research data were analysed with qualitative content analysis (Table II; detailed in Hilário et al. [33]). The methodological framework guiding the in-depth interviews and observations comes from ethnography [34–37]. We explored the potential of team ethnography [38,39], which meant the systematic sharing of observations from the field in regular meetings and used the guidance for in-depth interviewing and analysis [40,41].

**Table II.** Data sources and the details of methods for VAX-TRUST ethnographic research.

Research objectives	Data	Methods	Detailed methods
To analyse interactions between HCPs and parents and the position of HCPs in terms of vaccine hesitancy	Fieldnotes and reflection discussions from observations of encounters between HCPs and parents (numbers of observed encounters and observation hours may vary by country, depending on the encounters and sites observed; minimum two sites and (approximately 40–60 h)	Content analysis of fieldnotes, reflection within the team after field observations	Site selection: medical clinics, health organisations or agencies where vaccines are administered (e.g. paediatrician surgeries, child health clinics) in different socio-economic status neighbourhoods, including regions with low vaccine coverage rates Dimensions observed: description of the site, interaction between parents and HCPs, characteristics of participants (socio-economic status, cultural/religious background, cultural specificities) Special attention to be given to (a) good practices during the vaccine encounter and (b) critical interactions, such as absence or superficiality of information on side-effects, lack of empathy or not devoting enough time to the parents’ requests and needs
To gain information about HCPs’ perceptions and reflections on vaccines and vaccination programmes	Interviews with HCPs directly involved in childhood vaccination (approximately 30 per country)	Content analysis of semi-structured interviews	Recruitment strategies: direct invitation at vaccination sites, online discussion forums, suggestion of interviewed parents Heterogeneity of the sample is considered in terms of gender, work experience, age, attitude to vaccination Interview topics: own vaccine attitudes, parents’ perceptions on immunisation programmes and related health policies, encountering vaccine hesitancy at work, HCPs vaccine hesitancy, COVID-19 and vaccine hesitancy
To gain insights into the perceptions, attitudes and behaviour of vaccine-hesitant parents	Interviews with vaccine-hesitant parents with children in pre-school or of school age who have hesitated about at least one vaccination of their child (approximately 30 per country)	Content analysis of semi-structured interviews	Recruitment strategies: invitations through boards of ‘alternative’ schools, personal contacts, snowballing, university mailing lists, social media, organisations focusing on parenting, direct invitations, in-person selection at vaccination sites, local associations, flyers and posters Heterogeneity of sample is considered in terms of number of children, ethnic background and religion, level of education and socio-economic status Interview topics: attitudes and choices related to vaccinations, public debate on vaccination, opinions about mandatory vaccines

The HCP interviews were designed to gain information about the challenges that the HCPs face in meeting with vaccine-hesitant parents and to gain information about their considerations on the vaccination programmes and their own perceptions about vaccinations and vaccines. Through the vaccine-hesitant parent interviews, we gained an in-depth understanding of the reflections of the parents on the HCPs, healthcare authorities and healthcare system [26]; see more on our recruitment strategies in Hilário et al. [33]. This knowledge helped us to understand parental concerns regarding vaccines and how the parents see the broader societal situation concerning immunisation. We chose key informant interviews instead of group interview techniques because the latter may be inappropriate for exploring particularly sensitive issues that participants may feel uncomfortable about discussing in a group environment. Observations of HCP and parent interactions are of central importance in understanding the real-life encounter situations where vaccinations take place and for gaining knowledge about the technical and communicative practices, the power dynamics of the interaction, and the cognitive and emotional dimensions of the interaction [42].

## VAX-TRUST intervention design and evaluation

In all VAX-TRUST countries, we designed tailored, evidence-based interventions, which were educational sessions or reusable learning objects (see Table III). Considering the diversity of healthcare systems, HCPs previous education on vaccination and pre-existing interventions within the countries, the designed interventions were ‘complex’ [43,44]. The purpose of the interventions was to support HCPs and provide them with access to up-to-date and in-depth sociological research. Also, the interventions aimed to foster the professional self-reflexivity [45] of HCPs concerning the ways in which they approach vaccine-hesitant parents. Furthermore, the HCPs benefit from an opportunity to provide and receive support from peers facilitated by the interventions. Previous research in the healthcare domain shows that peer support is far from self-evident, although its benefits are well reported [46,47]. In the interventions’ design, we considered that HCPs may themselves be vaccine-hesitant, which may take many forms, from rejection of one or more vaccines to hesitating some, but taking them and giving them to their children [17,31,48–51].

**Table III.** Details of data and methods in VAX-TRUST intervention design and evaluation.

Research objectives	Data	Methods	Detailed methods
To map the most recent interventions targeted at HCPs to address vaccine hesitancy globally	Scientific articles	Systematic literature review from three databases (PubMed, Scopus and Embase)	Focus of analysis: analyse the tools and approaches of previous interventions Output of the review: a list of the most effective tools and approaches to address vaccine hesitancy
To study the latest interventions in the target regions	Scientific articles published in national journals not covered by international databases, internal publications of public or private organisations, technical reports of bodies or commissions, research projects and reports, proceedings or abstracts of congresses, conferences and seminars (including computer-based multimedia presentations), internal publications of local authorities, any resources and experiences produced by national and local institutions, research bodies, associations, interest groups, dissertations and doctoral theses, regulations and policy documents	Grey literature review	Criteria for selection: intervention directly targeted at HCPs, intervention targeted at reducing vaccine hesitancy or increasing vaccine uptake among HCPs or among their patients, intervention carried out in the target region Output of the review: narrative summary of the characteristics and evaluations of the interventions, with a focus on intervention, target and outcome types
To design and implement interventions aimed at increasing awareness on the complexity of vaccine hesitancy among HCPs involved in discussing childhood vaccines with parents	Intervention design documents, intervention materials (e.g. PowerPoint slides, groupwork materials, intervention report by implementers), reflections of the implementers, and feedback from interventions’ participants	Educational sessions (in-person or online) or reusable learning objects to HCPs (*n* = 50–100 per country), participants (nurses and medical doctors) either from the fieldwork sites of VAX-TRUST ethnographic study or HCPs involved in administering vaccines in the target regions	Implementers: social scientists who conducted the VAX-TRUST ethnographic study Intervention materials: based on VAX-TRUST situation analysis and ethnographic research Intervention design process: planning and description of the materials, core elements and content of the intervention Internal assessment: quantitative and qualitative pre- and post-assessment
To evaluate the suitability of the implemented interventions to effectively increase the awareness of HCPs of the complexity of vaccine hesitancy	Intervention design documents, intervention materials (protocols, measurements), evaluator’s observations, questionnaires, semi-structured interviews, document analysis	Evaluation of interventions by external evaluators (that are not part of the implementer team)	Evaluability analysis: re-check the overall rationale underpinning the interventions to make possible adjustments in the focus and expected outcomes before the implementation stage (clarity, plausibility, validity and reliability, contextualisation, complexity, agreement), inception of interventions (which indicators are selected and which measures are used) and implementation of interventions (specific mechanisms, available resources, process and outcomes assessment) Implementation analysis: a. Did the interventions produce the expected outcomes? If so, what can one learn to scale them up to other contexts (target regions and countries)? If no, which driving forces prevented them to happen and which strategies can overcome such limitations? b. What one can learn from the way the interventions were implemented with the different target audiences in different countries?

The interventions’ development started with literature reviews (Table III; more details in Lo Moro et al. [52]). The design process was grounded in the TIDieR (Template for Intervention Description and Replication) checklist [53] and the 6SquID (Six Steps in Quality Intervention Development) framework [54], both providing useful models for determining how to develop interventions to maximise their effectiveness. Three theoretical perspectives acted as the inspiration for the development of practical tools and core elements of the intervention: the social worlds framework [55], actor–network theory [56] and normalisation process theory [57]. No randomised controlled trial was used in the interventions’ design because the interventions were qualitative and iterative by nature.

Previous research has pointed to the need to increase the effectiveness of interventions aimed at addressing vaccine hesitancy [58]. The VAX-TRUST interventions were carefully evaluated to learn what works well in practice and why, and to enable transferring best practices across countries (Table III). The evaluation framework was grounded on the CDC Framework for Programmes Evaluation in Public Health [59], the WHO evaluation framework [60] and the international literature on evaluability assessments [61]. The evaluation team provided feedback on all steps of the intervention development, including planning, analysis and implementation. Providing feedback on the planning stages enabled improvements to take place before the interventions were implemented, which supported the full potential of the interventions being realised.

## Potential impact of the study

The VAX-TRUST approach, including the interventions designed to maximise the sharing of social scientific insights to healthcare settings, has the potential to: strengthen the expertise of HCPs to address vaccine hesitancy; benefit health care practices, health care education, and the development of materials and activities relating to vaccine hesitancy; and to provide evidence-based knowledge applicable for health policy-making in various European contexts.

To maximise the impact of the project, we worked closely with HCPs, medical and nursing educational institutions, and other immunisation stakeholders. VAX-TRUST includes the Finnish National Institute for Health and Welfare as a partner: in the other VAX-TRUST countries, we worked closely with national stakeholder advisory boards, consisting of important local or national stakeholders working on immunisation programmes. We also actively collaborated with the institutions with responsibility for designing and delivering medical and nursing education. Developing educational materials for medical and nursing students forms a part of VAX-TRUST exploitation activities. This engagement with HCPs, education institutions and health policy stakeholders could potentially make a concrete difference to understanding and influencing vaccine hesitancy in healthcare practices across various contexts. We focused not only on the current key stakeholders in the field, but also those of the future.

Currently, there are very few tested and evaluated interventions addressing vaccine hesitancy in Europe [17]. We developed, tested, implemented and evaluated tailored interventions for each VAX-TRUST country. With these interventions, VAX-TRUST has potential to increase HCP’s sensitivity towards understanding the perspective of hesitant parents. Simultaneously, the intervention may provide an opportunity for HCPs to reflect on their own relationship with vaccines.

By conducting VAX-TRUST research in seven countries, we captured the diversity in vaccine hesitancy in the European context. However, diversity was also present in our cultures of conducting sociological work combined with contributions from public health scholars. To address this notion, we invested a significant amount of attention to discussing, for example, the differing ethical guidelines and assumptions about dissemination. We formed our collaboration on the basis of mutual respect for different organisational, cultural and individual ways of working and communicating. This respect is essential for building successful and good collaboration practices in research teams [62]. Embracing the cultural diversity and shared learning within the consortium is particularly important when studying a topic as sensitive as vaccine hesitancy and it is a prerequisite for producing research outputs that can achieve a wide applicability and a sustainable impact.

Our objective in presenting the VAX-TRUST research approach is to encourage greater engagement across future and current projects using social science theory and methods. By outlining the design and the ethos of a project that is funded by the European Commission Health, Demographic Change and Wellbeing [63], and led by social scientists, we aim to encourage the enhanced integration of medical fields, social sciences and humanities. We hope that the VAX-TRUST approach reaches a broad spectrum of academic and practitioner audiences and serves as an example of social scientific research addressing complex societal challenges related to health and wellbeing. As such, this paper aims to increase the transparency of social scientific research and approach. Following the trend of social sciences to publish study design articles [64], we want to show how a multi-country, mixed-methods study was constructed. Laying out our research design serves as an example of how to translate complex public health issues into social scientific study and methods.
